# A smoking gun? Clonal expansion in response to cigarette exposure

**DOI:** 10.3389/fonc.2023.1252643

**Published:** 2023-08-03

**Authors:** Daniel I. Nathan, John Mascarenhas

**Affiliations:** Division of Hematology and Medical Oncology, Tisch Cancer Institute, Icahn School of Medicine at Mount Sinai, New York, NY, United States

**Keywords:** clonal hematopoeisis, JAK2 V617F, TET2 gene mutation, cigarette, ASXL1 mutations

Clonal hematopoiesis is an emerging entity of clinical and investigational concern. In this issue of Frontiers in Oncology, Ramanathan et al. present their data suggesting that exposure to cigarette smoke promotes clonal growth of *TET2 -/-* and increased survival of *JAK2* V617F cells. Mutations in *TET2* and *JAK2* are frequently observed in clonal hematopoiesis of indeterminate potential (CHIP), a phenomenon characterized by somatic mutations in hematopoietic stem and progenitor cells and associated with increased risk for myeloid malignancy, cardiovascular disease, and all-cause mortality (1, 2).

The authors further identify mechanisms which implicate inflammatory factors for clonal selection. Using a novel model for direct smoke inhalation, wild type mice were found to have increased inflammatory gene expression signatures and evidence of reactive oxidation and DNA stress in their bone marrow when exposed to cigarette smoke (CS) as compared to air. Further, the authors observed a corresponding quantitative decrease in the hematopoietic stem cell compartment, but not one that was not functionally apparent in re-population assays.

The relationship between inflammation and CHIP is well studied, with certain mutations, including *TET2* and *ASXL1*, propagating an inflammatory milieu that provides a selective growth advantage to existing CHIP clones (3, 4). Accordingly, CHIP is also enriched in cohorts of patients with systemic inflammatory diseases including vasculitis, rheumatoid arthritis, and gout (5–7). Of interest is the connection between the phenotypes of these patients and drivers of CHIP, and whether identifying high-risk individuals from selected cohorts could prevent adverse outcomes.

The finding that genotoxic and inflammatory insults resulting from CS exposure may promote CHIP further add to the growing body of knowledge into specific environmental drivers of clonal selection. CHIP enrichment has been identified in firefighters who assisted in recovery efforts at the World Trade Center (WTC) as compared with non-WTC firefighters (8). In mice, exposure to particulate matter from the WTC site proved mutagenic in a pattern similar to that of smoking, leading to the conclusion that the mass-exposure event from the WTC clean-up, in addition to age, may have provoked the high rates of observed CHIP amongst those firefighters.

It is noteworthy that while CHIP overall has been associated with smoking, specific gene associations have been limited to variants in *ASXL1*. The two genes modeled in the current study, *TET2* and *JAK2*, have not been found to be associated with smoking in large population studies from the UK BioBank and a cohort of cancer patients from Memorial Sloan Kettering Cancer Center (9, 10). A host of uncontrolled factors may be responsible for their absence in these unselected cohorts and there is still uncertainty regarding the causal nature of these findings. In one study of the UK BioBank applying Mendelian randomization analyses, smoking was not found to be causally linked to CHIP but did have an association with other structural somatic changes known as mosaic chromosomal alterations (11). However, in a separate analysis of the same cohort, smoking was found as causal for CHIP overall and for *DNMT3A* but not *TET2* mutant clones (12). While the benefit of smoking cessation is widespread across cancer prevention, the mechanistic implications of the present study for patients with known *TET2* or *JAK2* V617F CHIP may be potentially meaningful. Appropriately, the authors acknowledge the clinical significance of *ASXL1* CHIP with respect to smoking history and are actively exploring this connection with their models.

Currently, there are no guidelines for the surveillance or treatment of individuals with CHIP. And while there is a proliferation of clinics to monitor these individuals there is a dearth of interventions to prevent expansion of clones or progression to myeloid neoplasms. Clinical trials are underway to exploit the inflammatory dysregulation seen with CHIP in patients with somatic variants and unexplained cytopenias, a condition defined as clonal cytopenia of undetermined significance (CCUS) and of particular risk for neoplastic progression (13). So, for the time being, insight into mechanisms of clonal fitness is invaluable to develop real world best-practices for monitoring and advising these patients. Given the present findings that CS exposure facilitates the survival of clones carrying two common CHIP mutations, smoking cessation should be considered an important intervention in our scant armamentarium to prevent progression to myeloid malignancy. Further clinical investigation and prospective trials are sorely needed to build on these key findings.

## Author contributions

DN conceived and wrote the first draft and JM edited and revised the manuscript.

## References

[1] Jaiswal S Fontanillas P Flannick J Manning A Grauman PV Mar BG. Age-related clonal hematopoiesis associated with adverse outcomes. N Engl J Med (2014) 371(26):2488–98. doi: 10.1056/NEJMoa1408617 PMC4306669 25426837

[2] Jaiswal S Natarajan P Silver AJ Gibson CJ Bick AG Shvartz E. Clonal hematopoiesis and risk of atherosclerotic cardiovascular disease. N Engl J Med (2017) 377(2):111–21. doi: 10.1056/NEJMoa1701719 PMC6717509 28636844

[3] Cai Z Kotzin JJ Ramdas B Chen S Nelanuthala S Palam LR. Inhibition of inflammatory signaling in tet2 mutant preleukemic cells mitigates stress-induced abnormalities and clonal hematopoiesis. Cell Stem Cell (2018) 23(6):833–49 e5. doi: 10.1016/j.stem.2018.10.013 30526882 PMC6317370

[4] Avagyan S Henninger JE Mannherz WP Mistry M Yoon J Yang S. Resistance to inflammation underlies enhanced fitness in clonal hematopoiesis. Science (2021) 374(6568):768–72. doi: 10.1126/science.aba9304 34735227

[5] Agrawal M Niroula A Cunin P McConkey M Kovalcik V Kim PG. Tet2-mutant clonal hematopoiesis and risk of gout. Blood (2022) 140(10):1094–1103. doi: 10.1182/blood.2022015384PMID-35714308 PMC9461470 35714308

[6] Savola P Lundgren S Keränen MAI Almusa H Ellonen P Leirisalo-Repo M. Clonal hematopoiesis in patients with rheumatoid arthritis. Blood Cancer J (2018) 8(8):69. doi: 10.1038/s41408-018-0107-2 30061683 PMC6066480

[7] Arends CM Weiss M Christen F Eulenberg-Gustavus C Rousselle A Kettritz R. Clonal hematopoiesis in patients with anca-associated vasculitis. Haematologica (2019) 105(6):e264–267. doi: 10.3324/haematol.2019.223305 PMC7271609 31582546

[8] Jasra S Giricz O Zeig-Owens R Pradhan K Goldfarb DG Barreto-Galvez A. High burden of clonal hematopoiesis in first responders exposed to the world trade center disaster. Nat Med (2022) 28(3):468–71. doi: 10.1038/s41591-022-01708-3 PMC9394171 35256801

[9] Dawoud AAZ Tapper WJ Cross NCP. Clonal myelopoiesis in the uk biobank cohort: asxl1 mutations are strongly associated with smoking. Leukemia (2020) 34(10):2660–72. doi: 10.1038/s41375-020-0896-8 32518416

[10] Bolton KL Ptashkin RN Gao T Braunstein L Devlin SM Kelly D. Cancer therapy shapes the fitness landscape of clonal hematopoiesis. Nat Genet (2020) 52(11):1219–26. doi: 10.1038/s41588-020-00710-0 PMC7891089 33106634

[11] Levin MG Nakao T Zekavat SM Koyama S Bick AG Niroula A. Genetics of smoking and risk of clonal hematopoiesis. Sci Rep (2022) 12(1):7248. doi: 10.1038/s41598-022-09604-z 35508625 PMC9068754

[12] Kar SP Quiros PM Gu M Jiang T Mitchell J Langdon R. Genome-wide analyses of 200,453 individuals yield new insights into the causes and consequences of clonal hematopoiesis. Nat Genet (2022) 54(8):1155–66. doi: 10.1038/s41588-022-01121-z PMC9355874 35835912

[13] Niroula A Sekar A Murakami MA Trinder M Agrawal M Wong WJ. Distinction of lymphoid and myeloid clonal hematopoiesis. Nat Med (2021) 27(11):1921–7. doi: 10.1038/s41591-021-01521-4 PMC8621497 34663986

